# Editorial: Ethical and legal implications of artificial intelligence in public health: balancing innovation and privacy

**DOI:** 10.3389/fmed.2026.1885165

**Published:** 2026-05-28

**Authors:** Giovanna Ricci, Paolo Bailo, Filippo Gibelli

**Affiliations:** Section of Legal Medicine, School of Law, University of Camerino, Camerino, Italy

**Keywords:** accountability, artificial intelligence, fairness, health data governance, human oversight, privacy, public health, public trust

## Introduction

This Research Topic shows that AI in public health cannot be regulated in terms of an exchange between innovation and privacy alone. In all the articles, which range from preparedness among clinicians, explainability, data regulation, equity, community engagement, and large language models, the common theme is how institutions should regulate AI from inception through to its life cycle stages. From data collection and modeling, validation, implementation, monitoring, and correction, the key questions that arise are about performance and quality of data, transparency, justice, safety, accountability, professionalism, patient autonomy, governance, and trust. The larger context is important since the AI system in public health does more than optimizing current methods and processes; it also involves distribution of epistemic power and responsibility and the representation of people and communities.

## Thematic synthesis of the Research Topic

The first theme relates to legitimacy that goes beyond the issue of good performance metrics. Perrella et al., Nasir et al., Giorgetti et al., and Jha et al., all touch upon this theme. While their approaches are distinct—focusing on preparedness and regulation, critical analysis of practices of data marginalization, explainability and liability, and the governance of foundational models for medical imaging, respectively—the authors agree on one important point. To be able to deliver value to the public health sphere, an AI solution should be developed by professionals, well-documented, interpretable, and have clearly delineated routes for institutional accountability.

Health data governance, consent, secondary uses, and commodification constitute the next group of themes. Al Qwaid, Liang, Shaikh et al., and Fan et al. demonstrate that besides legal permission to use data, the success of responsible AI development relies on whether data practices are understandable, appropriate, protected from unauthorized access and use, and consistent with the public interest. Taken together, the mentioned authors address issues such as ownership, consent, privacy-preserving design, fairness-aware training, traceability, and responsibility throughout the entire research and development cycle. As a common implication of their findings, one can argue that the ethical foundation of datafication becomes unstable whenever it becomes either opaque or exploitative.

A third theme relates to issues of equity, safety, and public trust. Balakrishnan et al., Li et al., Rice et al., and Townsend et al. reveal how bias can manifest itself in clinical and workforce-related contexts, how diagnostic error is intertwined with opacity and lack of accountability, the importance of participant-centered and historically informed governance for Indigenous peoples' health care systems, and the nature of public concern extending beyond malfunctions to social, psychological, and regulatory aspects. In public health care systems, it is unjustifiable to accept average benefits alongside the preventable concentrations of risk. In other words, fairness should be integrated into the processes of representation and design prospectively.

The question of professional responsibility and human oversight emerges yet again in multiple contributions. Nofal et al. make clear that preparing staff for using AI is more than just implementing a solution, whereas it is rather about proper decision-making related to escalation, documentation, etc. Holmes et al. and Xiong et al., in their way show how generation of synthetic data challenges patient agency, communicative reliability, and trustworthiness: in synthetic/hybrid environments, the link between personal experience and organizational expertise can be weakened, while some recommendations generated by AI tools can prove themselves to be ethically problematic. Human oversight thus cannot be reduced to post facto human presence.

To summarize, the *Research Topic* highlights several operational requirements for AI solutions in the context of public health care. Namely, before deployment, institutions need to clarify intended use, targeted population groups, data provenance and validation criteria, duties and responsibilities, and escalation pathways; during deployment—monitor performance, possible biases, misuse, drifts, professional reliance, and patient communication; and, after deployment—create audit trails, incident reports, post-market correction, and means of redress. Such measures are medico-legal in nature since they ensure that once harm arises due to AI tools, it will become possible to trace who designed, purchased, authorized, relied on, monitored, and corrected the system at hand.

## Toward accountable life-cycle governance

This transition from abstraction into practical governance is buttressed by the existing regulatory regime. GDPR and EU AI Act offer critical foundations for lawful activity, data protection, risk management, transparency, human control, and post-market monitoring (1, 2). The EHDS and DGA contribute additional sector-specific and infrastructural elements via the linkage of electronic health data, interoperability, re-use, public interest objectives, and confidence in data sharing (3, 4). WHO, UNESCO, OECD guidelines, and the Framework Convention of the Council of Europe bring human rights, public benefit, democratic accountability, and institutional accountability into the scope of AI governance (5–9). EDPB and EDPB-EDPS publications are especially relevant, since they address the relationship between AI and legal basis for processing, anonymization, legitimate interests, data protection by design, and data protection vs. AI regulation (10–12). However, as the present Research Topic demonstrates, compliance is not the only pillar of trustworthy public health AI. Ethical acceptability hinges upon the capacity of those impacted by the AI process to comprehend, challenge, and place trust in such decisions, and of organizations to monitor, repair, and, if required, suspend such operations following deployment.

## Conclusion

Collectively, the contributions indicate that the use of AI in public health should not be managed in terms of the traditional tension between innovation and privacy. Rather, a better perspective is that of life-cycle governance, where considerations such as data quality, privacy, transparency, equity, safety, accountability, professionalism, patient autonomy, organizational oversight, and public trust must be viewed as interlocking prerequisites for legitimacy. In this sense, the primary contribution of this Research Topic is both theoretical and practical. It demonstrates that responsible AI in public health requires proactive, multi-disciplinary, and verifiable forms of governance that can be sustainably implemented at all stages.
